# Response to Martinez-Novack *et al*. Comments on Hynes *et al*. Prevalence of Marijuana Use among University Students in Bolivia, Colombia, Ecuador, and Peru. *Int. J. Environ. Res. Public Health*, 2015, *12*, 5233-5240

**DOI:** 10.3390/ijerph120911721

**Published:** 2015-09-17

**Authors:** Marya Hynes, Maria Demarco, Juan Carlos Araneda, Francisco Cumsille

**Affiliations:** 1 Inter-American Observatory on Drugs, Inter-American Drug Abuse Control Commission (CICAD), Organization of American States (OAS), 1889 F Street NW, Washington, DC 20006, USA; E-Mails: mdemarco@oas.org (M.D.); fcumsille@oas.org (F.C.); 2 Global SMART Programme (Latin America), United Nations Office on Drugs and Crime, 1889 F Street NW, Washington, DC 20006, USA; E-Mail: jaraneda@oas.org

We have read with great interest the Comments related to the article entitled “Prevalence of marijuana use among university 20 students in Bolivia, Colombia, Ecuador and Peru” and appreciate the readers’ feedback [1]. 

For our study “Prevalence of marijuana use among university students in Bolivia, Colombia, Ecuador and Peru” [2], a sample size was calculated to meet certain conditions of accuracy and reliability. We doubled this sample size to reach 50 percent response rates from the original sample. Based on our calculations, our target response rate was 50 percent.

Response rates in our study are shown in Table 1. Response rates were 43.26 percent of the original sample in 2009 and 31.94 percent in 2012. Response rates in the survey did not reach the target of 50 percent, which is indeed a limitation of the study. The overall response rates were 86.52 percent of the expected sample in 2009 and 63.88 percent in 2012.

We present the weighted estimates, which were included in the original article, in Table 2. In addition, we created Table 3 with unweighted estimates of the prevalence of marijuana use in Andean countries. While readers might find unweighted estimates informational, estimates for the weighted sample are more precise because weighting the data tends to inflate sample size [3].

We would like to thank the readers for their comments as this will help us to improve our future analyses.

**Table 1:** Response rates from original and expected samples in Bolivia, Colombia, Ecuador, and Peru, 2009 and 2012.

	Response Rates (Original Sample)		Response Rates (Expected Sample)	
	2009	2012	2009	2012
Bolivia	27.83%	20.05%	55.66%	40.10%
Colombia	67.75%	54.30%	135.51%	108.60%
Ecuador	31.18%	24.16%	62.37%	48.32%
Peru	46.28%	29.24%	92.56%	58.47%
**TOTAL**	**43.26%**	**31.94%**	**86.52%**	**63.88%**

**Table 2:** Prevalence of marijuana use in Andean countries (weighted) (Differences in prevalence between 2009 and 2012 were statistically significant (*p* < 0.01) for all countries, in the overall population and among males. Differences in prevalence for females were only significant in Colombia and Ecuador).

		Bolivia		Colombia		Ecuador		Peru	
		2009	2012	2009	2012	2009	2012	2009	2012
**Lifetime**	Males	11.09	19.65	32.83	39.03	17.25	32.37	10.51	16.88
	Females	3.76	6.44	19.27	24.35	6.18	13.10	5.61	6.57
	**Total**	**7.49**	**11.97**	**26.41**	**31.16**	**11.41**	**21.94**	**8.40**	**11.58**
**Past year**	Males	2.66	5.06	14.55	19.86	6.88	12.64	3.36	6.02
	Females	1.40	2.27	8.15	10.81	2.24	5.93	2.46	2.65
	**Total**	**2.04**	**3.44**	**11.51**	**15.01**	**4.43**	**9.00**	**2.97**	**4.29**
**Past month**	Males	0.94	2.45	6.76	10.26	2.10	5.52	1.08	2.66
	Females	0.56	0.73	3.61	4.44	1.30	2.10	0.91	0.63
	**Total**	**0.76**	**1.45**	**5.27**	**7.14**	**1.68**	**3.67**	**1.00**	**1.62**

**Table 3:** Prevalence of marijuana use in Andean countries (unweighted) (Differences in prevalence between 2009 and 2012 were statistically significant (*p* < 0.01) for all countries, in the overall population and among males. Differences in prevalence for females were only significant in Colombia and Ecuador.)

		Bolivia		Colombia		Ecuador		Peru	
		2009	2012	2009	2012	2009	2012	2009	2012
**Lifetime**	Males	18.87	18.65	23.41	35.82	23.41	40.51	13.80	16.75
	Females	8.71	7.14	13.47	22.23	7.13	18.58	7.52	7.61
	**Total**	**13.04**	**12.09**	**18.08**	**28.63**	**15.05**	**29.19**	**10.59**	**12.14**
**Past year**	Males	6.06	5.96	10.58	18.38	7.76	19.05	5.02	6.61
	Females	3.47	2.77	5.90	9.82	2.96	9.32	3.30	3.09
	**Total**	**4.69**	**4.14**	**8.07**	**13.80**	**5.30**	**14.03**	**4.14**	**4.83**
**Past month**	Males	2.34	2.59	4.81	9.51	3.32	9.11	1.73	2.69
	Females	1.14	1.00	2.34	3.97	1.14	3.20	1.28	0.89
	**Total**	**1.71**	**1.68**	**3.49**	**6.55**	**2.20**	**6.06**	**1.50**	**1.78**
